# Cementum regeneration strategies: Insights from development and periodontal microenvironment

**DOI:** 10.3389/fcell.2025.1685609

**Published:** 2025-10-01

**Authors:** Huiyi Wang, Liu Yang, Xin Huang, Li Ma, Chuan Wang, Xiaoxuan Wang, Zhengguo Cao

**Affiliations:** ^1^ State Key Laboratory of Oral and Maxillofacial Reconstruction and Regeneration, Key Laboratory of Oral Biomedicine Ministry of Education, Hubei Key Laboratory of Stomatology, School and Hospital of Stomatology, Wuhan University, Wuhan, China; ^2^ Department of Periodontology, School and Hospital of Stomatology, Wuhan University, Wuhan, China

**Keywords:** dental cementum, cementum development, mineralization, periodontal microenvironment, regeneration

## Abstract

Cementum, a mineralized tissue covering the root surface of the tooth, maintains tooth stability through providing support for the attachment of periodontal ligament fibres, and protects the pulp from external damage *via* acting as a barrier against microbial invasion and destruction. Notably, both physiological and pathological factors may lead to the destruction of this vulnerable tissue, thus impeding its function. However, the intricate periodontal microenvironment, which consists of host cells, microbial communities, and metabolites, presents challenges to cementum regeneration. In addition, there remains a debate regarding whether the cellular origin of cementum is derived from mesenchymal cells or epithelial cells. Due to the limitations of traditional regenerative surgeries in achieving complete cementum regeneration, researchers are exploring new strategies based on cementum development and the periodontal microenvironment. Our group has revealed the crucial regulatory mechanisms in cementoblast differentiation and developed engineering materials for cementum regeneration. Drawing upon the latest research on cementum development and regeneration, alongside the comprehensive studies undertaken by our research group over the years, this review systematically consolidates current knowledge on cementum development and the regulatory functions of the periodontal microenvironment. It emphasizes mechanisms such as metabolic reprogramming, epigenetic modifications, and immune-stem cell interactions. Furthermore, the review seeks to provide innovative, target-oriented insights for strategies aimed at cementum regeneration, grounded in the understanding of cementum development and the periodontal microenvironment.

## 1 Introduction

Cementum, interposed between the tooth dentin and periodontal ligament, can be described as the mineralized and avascular tissue that covers the roots of teeth. Cementum attaches fibers of the periodontal ligament to the root surface, serving essential adaptive and reparative purposes. It functions as a barrier to confine epithelial growth and hinder bacterial evasion (Arzate et al., 2015). A highly adaptable cementum is vital for sustaining occlusal relationships and ensuring the root surface remains intact. However, the theories concerning the development of cementum remain conflicting, largely due to the complex cellular dynamics during root formation and limitations in research methodologies. One theory is the classical mesenchymal hypothesis, and another theory is the alternative epithelial hypothesis. Classical mesenchymal hypothesis states that cementoblasts derive from the mesenchymal dental follicle (Cho and Garant, 1988). Nevertheless, the alternative epithelial hypothesis suggests an epithelial contribution to cementogenesis *via* epithelial-mesenchymal transition (EMT) (Zeichner-David, 2006). Our research group was the first to reveal that deleting *Osterix* conditionally in mesenchymal cells results in reduced cellular cementum formation, indicating its mesenchymal origin (Cao et al., 2012).

However, the cementum is vulnerable to external stimuli. The breakdown of cementum can be caused by periodontitis, apical periodontitis, orthodontic-induced root resorption and other factors (Fuss et al., 2003). The periodontal microenvironment, shaped by stem cells, immune cells, microbial communities, and metabolites, constitutes a critical factor regulating periodontal regeneration. For instance, microbial dysbiosis alters local metabolite profiles, disrupting osteoimmunological balance and accelerating resorption, while anti-inflammatory metabolites may counteract these effects. Besides, recent advances in metabolomics have unveiled the underappreciated role of metabolites (e.g., lipids, lactate) in regulating cementoblast mineralization and regeneration. In the process of cementum regeneration, a variety of mechanisms such as signal transduction, cell metabolism and epigenetic modifications are involved. Our group has revealed that lncRNAs, histone modifications, DNA methylation, and post-translational protein modifications play crucial regulatory roles in cementoblast mineralization and modulation of periodontal microenvironment.

With the deepening basic and clinical research, many strategies have been proposed for promoting cementum regeneration. Multiple regenerative methods have been developed, such as guided tissue regeneration (GTR) with various membranes, root surface biomodification and other techniques (Bottino et al., 2012; Shibli et al., 2022). Nevertheless, the use of GTR membranes for periodontal regeneration is partially effective. For instance, some GTR membranes can affect cell colonization on exposed root surfaces. Additionally, they may collapse in larger defects during tissue regeneration. Most commercially available GTR membranes also have limited osteogenicity and lack anti-inflammatory and antimicrobial properties, posing challenges for cementum formation and new periodontal attachment (Ma and Yan, 2023). To regenerate the whole periodontium, multiple materials targeting components in the periodontal microenvironment are now being developed, including stem cells, extracellular vesicles, and biomaterial scaffolds. These regeneration strategies synergistically enable the delivery of bioactive agents such as matrix proteins, RNA/miRNAs, and metabolites, which augment periodontal regeneration by modulating cellular dynamics and promoting microenvironmental homeostasis (Abedi et al., 2022).

This review synthesizes contemporary evidence on cementum development, emphasizing the dynamic interactions within the periodontal microenvironment, which includes host cells, microbial communities, and metabolites, in both physiological and pathological contexts. Additionally, we elucidate the mechanisms underlying cementum regeneration, encompassing signal transduction, metabolic reprogramming, and epigenetic regulation. Moreover, this review integrates developmental biology with microenvironmental dynamics, offering a comprehensive perspective on cementum regeneration. We particularly highlight recent advancements in microenvironment-inspired strategies by bridging fundamental mechanisms with translational applications, such as engineered extracellular vesicles, metabolic modulation, and epigenetic interventions, which hold promise for achieving functional and biomimetic cementum restoration.

## 2 Development pattern of cementum

### 2.1 Types of cementum

**FIGURE 1:**
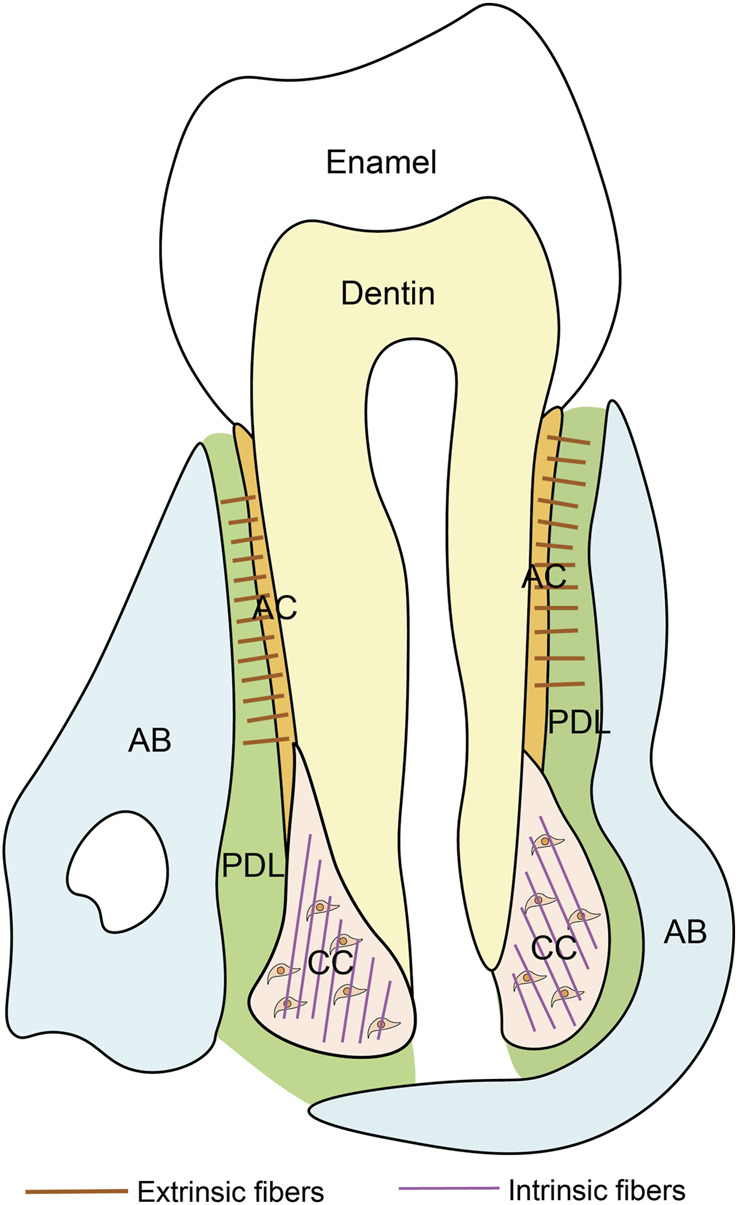
Schematic of the tooth. PDL, periodontal ligament; AB, alveolar bone; AC, acellular cementum; CC, cellular cementum.

Cementum, a vital component of teeth, is histologically classified into two types: into two types: acellular cementum (or acellular extrinsic fiber cementum) and cellular cementum (or cellular intrinsic fiber cementum) (Figure 1) (Bosshardt and Selvig, 1997). They exhibit distinct differences in formation timing, structural characteristics, and functions (Table 1).

**TABLE 1:** The characteristics of acellular cementum and cellular cementum.

Characteristics	Acellular cementum	Cellular cementum
Forming time	During root elongation	After full elongation of tooth root
Containing cells	No	Yes
Thickness	Thin	Thick
Structure	Containing collagen fibers and non-collagenous proteins The insertion of Sharpey’s fibers from the periodontal ligament.	Containing mineralized matrix The intrinsic fibers of the cellular cementum are aligned parallel to the root surface.
Location	Coronal and mid-portions of the root	Apical and interradicular portions
Function	Providing periodontal fibers attach and stabilizing teeth.	Repairing tissues; adaptation functions.

#### 2.1.1 Acellular cementum

The acellular cementum is a thin mineralized tissue covering the coronal and mid-portions of the root. Its thickness, varying between 50 and 200 μm, increases with age. Developmentally, acellular cementum is formed during root elongation (Foster, 2012). It is devoid of cells and consists of collagen fibers and non-collagenous proteins as organic matrices, both of which are entirely mineralized. A notable feature of acellular cementum is the embedding of collagen fibers from the periodontal ligament, known as Sharpey’s fibers. The extrinsic fibers are compactly organized and set nearly at a perpendicular angle to the root surface. Functionally, it is responsible for attaching the tooth to the surrounding periodontal ligament and represents the ideal target for cementum regeneration (Yamamoto et al., 2016).

#### 2.1.2 Cellular cementum

The cellular cementum is thicker than acellular cementum, forming on the apical and interradicular portions. At the forming root tip, the cellular cementum deposition starts almost concurrently with dentin formation, postnatally forming after full elongation of the tooth root. The bone-like cellular cementum often contains cells and mineralized matrix, like extrinsic fibers. The density of these fibers varies across individuals. In cellular cementum, the intrinsic fibers are oriented parallel to the root surface and form nearly right angles with the extrinsic fibers (Schroeder, 1993). Cellular cementum is thought to be involved in the teeth movement and adjustment of occlusion (Luczak et al., 2025).

### 2.2 Cellular origin of cementum

**FIGURE 2:**
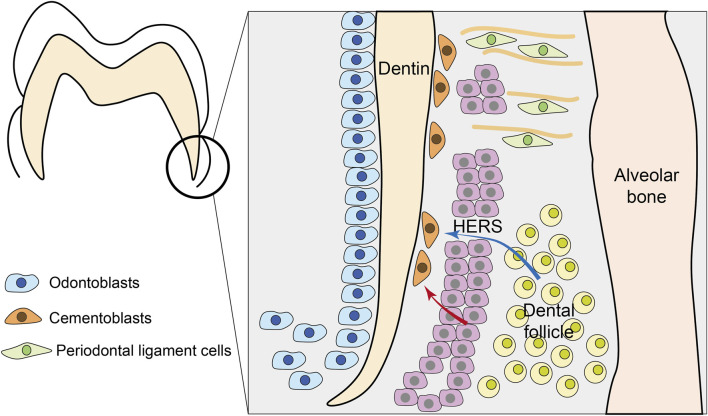
Development of cementum. During tooth root formation, Hertwig’s epithelial root sheath (HERS) fragments to allow interaction between epithelial and mesenchymal compartments. Two primary theories explain cementoblast origin: (1) the classical mesenchymal hypothesis (blue arrows) where dental follicle mesenchymal cells differentiate into cementoblasts; and (2) the epithelial hypothesis (red arrows) where HERS-derived cells undergo epithelial-mesenchymal transition (EMT) to become cementoblasts. Besides, mesenchymal PDL cells can also participate in cementum formation.

Cementum development initiates when both epithelial cells of Hertwig’s epithelial root sheath (HERS) and mesenchymal cells of the dental follicle are in proximity to the developing root surface (Figure 2). Cementum is formed by cementoblasts, yet the origin of cementoblasts is still controversial. Along with the ongoing advancement of research methodologies, the comprehension of the origin of cementoblasts has been steadily deepening, which can be divided into two theories. One is the classical mesenchymal hypothesis, and the other is the alternative epithelial hypothesis.

The prevailing classical theory posits that cementoblasts originate from the dental follicle proper and perifollicular mesenchyme. Around the 1950s, Paynter et al. collected mandibular bone slices of rats aged 10–101 days and found through immunohistochemical staining that when the tooth crown development was basically completed, the enamel organ began to proliferate and to form the epithelial root sheath. Then, the sheath broke up to allow the cells in contact with the root, followed by the innermost layer of cementum in contact with the dentin (Paynter and Pudy, 1958). Diekwisch et al., through *in situ* hybridization and immunolocalization techniques, demonstrated that mesenchymal cells derived from the dental follicle penetrate the bilayer structure of HERS and deposit the initial cementum (Diekwisch, 2001).

And others demonstrate that HERS cells themselves can directly acquire cementogenic potential *via* epithelial-mesenchymal transition (EMT). The alternative epithelial hypothesis was first proposed in an *in vitro* study by Thomas. He found that the cultured epithelial sheath cells transformed into mesenchymal cementoblasts (Thomas, 1995). *In vivo* studies have revealed that certain cementoblasts and cementocytes in the root regions of rats and mice exhibit keratin immunoreactivity, which is a marker of epithelial cells. These cells are considered to be epithelial sheath cells undergoing EMT during that period, as demonstrated by their capacity to form mineralized nodules *in vitro* (Bosshardt, 2005; Huang et al., 2009; Li et al., 2019).

Additionally, some researches indicate that cementoblasts forming acellular cementum and cellular cementum have different cellular origins. Distinct immunophenotypic profiles and cytokeratin expression patterns suggest that cementoblasts forming cellular/reparative cementum originate from dental mesenchymal progenitors, whereas acellular cementum-forming cells likely derive from epithelial-derived precursors (Zeichner-David et al., 2003).

Merely relying on the morphology observation of cementum development in mice or rats at different postnatal weeks is insufficient to address the issue of the developmental origin of cementoblasts. Thus, researchers have established conditional gene knockout mouse models using the Cre/LoxP system to investigate cementum development. Our group demonstrated that through mesenchymal cell-specific knockout of *Osterix* (*Osx*), a zinc finger-containing transcription factor, the number of cementoblasts was decreased (Cao et al., 2012). Conditional *Alpl* ablation *via* using *Col1a1*-Cre mice caused absence or reduction of acellular cementum (Foster et al., 2017). These findings lend support to the classical mesenchymal hypothesis.

The utilization of cell lineage tracing technology has significantly enhanced the understanding of the origins of cementum. Chai et al. reported that HERS cells participate in the formation of cellular cementum and may differentiate into cementoblasts *via* epithelial-specific knockout of *R26R* using *K14*-cre (Huang et al., 2009). This result supports the alternative epithelial hypothesis. In recent years, increasing evidence derived from cell lineage tracing technology has corroborated the classical theory that cementum originates from dental mesenchymal progenitors within the PDL. Gli1^+^ progenitor cells are reported to contribute to both acellular and cellular cementum (Xie et al., 2021). In addition, Axin2^+^ mesenchymal PDL cells are key progenitor cell sources that play a crucial role in rapid cementum growth, and both cellular and acellular cementum share the same progenitor cell sources (Xie et al., 2019; Ma et al., 2023).

### 2.3 Regulation of cementum matrix apposition

Following initial cementogenesis, cementum matrix apposition begins and is tightly regulated by specific transcription factors and signaling molecules (Somerman et al., 1991; Foster et al., 2006). Table 2 summarizes the regulatory effects of different signaling molecules on cementum matrix deposition. Our group first reported that Osterix (OSX) is a central molecule controlling cellular cementum formation, as conditional deletion of *Osx* causes a marked decline in the development of cellular cementum (Cao et al., 2012). Mechanistically, we found that Osx drives cementoblast differentiation through DKK1-mediated inhibition of Wnt/β-catenin signaling (Cao et al., 2015). Additionally, TGF-β signaling has been demonstrated to promote cellular cementum matrix apposition by activating Osx expression through a Smad-dependent pathway (Choi et al., 2016), indicating that the TGF-β/Smad/Osx axis is crucial for cementoblast differentiation and function.

**TABLE 2:** The regulation of cementum matrix deposition by different molecules.

Molecules	*In vivo* or *in vitro*	Results	References
Osterix	*In vivo*: *Osx* cKO mice. *In vitro*: Murine cementoblasts.	Positively regulating cellular cementum formation.	Cao et al. (2012)
TGF-β	*In vivo*: *OC* ^*Cre*^ *Tgfbr2* ^*fl/fl*^ mice. *In vitro*: Murine cementoblasts.	Promoting cellular cementum matrix apposition by activating *Osx* expression.	Choi et al. (2016)
Wnt/β-catenin signaling pathway	*In vivo: OC-Cre:Catnb(+/lox(ex3)) mice.* *In vivo: Axin2* ^*CreERT2/+*^ *; R26R* ^*DTA/+*^ *mice.* *In vivo: Gli1* ^*CreERT2/+*^ *; β-catenin* ^*flox(Ex3)/+*^ *; R26R* ^*tdTomato/+*^ *(CA-β-cat mice).*	Excessive Wnt/b-catenin signaling induces cementum hyperplasia. Activation of β-catenin in Axin2^+^-mesenchymal periodontal ligament cells are responsible for mineral matrix apposition. Conditional deletion of β-catenin in Gli1^+^ -mesenchymal progenitors cells lead to a decrease in postnatal cementum growth.	Bae et al., 2013; Xie et al., 2019; Xie et al., 2021
Wnt1	*In vivo*: *Wnt1*-transgenic (Wnt1Tg) mice. *In vitro*: Murine cementoblasts.	Enhancing both acellular and cellular cementum formation.	Nottmeier et al. (2021)
Pyrophosphate (PPi) and inorganic phosphate (Pi)	*In vivo*: *Alpl* KO mice. *In vitro*: Murine cementoblasts.	Negatively regulating acellular cementum volume.	Chu et al. (2020)
Ank and Enpp1	*In vivo*: *Ank and Enpp* ^*−/−*^ mice. *In vitro*: Murine cementoblasts.	Causing hypercementosis *via* regulating Pi/PPi ratio.	Foster et al. (2012)
Phex	*In vivo*: XLH/Hyp mice.	Resulting in reduced formation of acellular cementum and hypomineralized cellular cementum.	Coyac et al. (2017)
Phosopho1	*In vivo*: *Phospho1* ^*−/−*^ mice.	Enhancing cellular cementum deposition.	Zweifler et al. (2016)
Mdm2	*In vivo*: *Dmp1-Cre; Mdm2* ^*flox/flox*^ mice *In vitro*: Murine cementoblasts.	Maintaining cellular cementum volume.	Tian et al. (2025)
BMP2, CAP, CEMP1	*In vitro*: human periodontal ligament-derived cells.	Initiating mineralization and cementogenesis.	Hong et al. (2022)
BSP	*In vivo*: Bsp^−/−^ mice	Positively regulating acellular cementum mineralization.	Foster et al. (2013)
BMP1	*In vivo*: *Bmp1/Tll1* double knockout mice.	Maintaining appropriate levels of procollagen I for cementum homeostasis.	Wang et al. (2017b)
Ptx3	*In vivo*: *Ptx3* ^*−/−*^ mice. *In vitro*: Murine cementoblasts.	Positively regulating the formation of both acellular and cellular formation.	Wang et al. (2024a)

Wnt/β-catenin signaling pathway has been widely recognized to play a central role in cementum matrix apposition. Activating Wnt/β-catenin signaling by constitutively expressing β-catenin in OC-expressing cementoblasts or Gli1/Axin2^+^ lineage cells induces cellular cementum hyperplasia (Bae et al., 2013; Xie et al., 2019; 2021). Moreover, overexpression of Wnt1, a canonical Wnt ligand, enhances both acellular and cellular cementum formation by stimulating cementoblast proliferation (Nottmeier et al., 2021). Sclerostin (Sost), a secreted Wnt antagonist, is expressed by cementocytes both *in vivo* and *in vitro* (Jäger et al., 2010). Initial studies reported that Sost deletion in mice leads to a 1.5-fold increase in cellular cementum deposition along with enhanced alveolar bone formation by upregulating Wnt/β-catenin signaling (Li et al., 2008; Kuchler et al., 2014). However, a recent study found no significant effect on cementum formation in Sost knockout mice (Phanrungsuwan et al., 2025), indicating that cementum is less sensitive to the absence of Sost compared with bone. During the mineralization of cementum, β-catenin and Osx interact in the process of cementum formation. Wnt/β-catenin signaling positively regulates Osx expression, promoting cementoblast differentiation and the secretion of cementum matrix (Choi et al., 2019). Interestingly, it is also reported that Osx can modulate Wnt pathway activity by regulating the expression of Tcf/Lef family members to promote cementogenesis (Choi et al., 2017). These studies reveal the importance of Wnt/β-catenin/Osx signaling axis in cementum formation. Wnt signaling also interacts with other signaling pathways to control cementum matrix apposition. It is reported that sustained Hedgehog activity in cementoblasts negatively regulates postnatal cementum matrix apposition in a Wnt/catenin/Osx-dependent manner (Choi et al., 2020).

Foster and Somerman discovered that another important regulator of cementum matrix apposition is pyrophosphate (PPi) and inorganic phosphate (Pi) homeostasis, controlled by key PPi regulators (Chu et al., 2020). Biomineralization is governed by the dynamic balance between inorganic phosphate (Pi), a hydroxyapatite precursor, and PPi, its potent physiological inhibitor (Orriss et al., 2016; Thumbigere-Math et al., 2018). This equilibrium is maintained by three key regulators: ANK/Ank (mediating PPi transport), ENPP1/Enpp1 (generating extracellular PPi), and TNAP/Alpl (hydrolyzing PPi to Pi) (Millán, 2006; Szeri et al., 2020). Disturbances in the Pi and PPi ratio greatly affect the formation and physiological maintenance of cementum. In genetic models, Ank or Enpp1 deficiency causes hypercementosis due to elevated Pi/PPi, while *Alpl* loss disrupts cementum formation through reduced Pi/PPi (Foster et al., 2012; Nagasaki et al., 2021). At the cervical root surface, cementoblasts precisely modulate PPi levels through differential *Ank* and *Enpp1* expression. Both *Ank* and *Enpp1* knockout models exhibit excessive acellular cementum formation, confirming their shared role in mineralization control. *Enpp1*-deficient mice particularly accumulate non-collagenous proteins in ectopic cervical cementum, a process mediated by enhanced β-catenin activity and subsequent Osx upregulation (Choi et al., 2019; 2022). The phosphate-regulating neutral endopeptidase gene (*Phex*), expressed exclusively in cementocytes and not cementoblasts, is essential for proper cementum formation. Intriguingly, *Phex* deficiency results in reduced formation of acellular cementum and hypomineralized cellular cementum (Biosse Duplan et al., 2017; Coyac et al., 2017; Zhang H. et al., 2020). The phenotype of acellular cementum may result from hypophosphatemia since it is sensitive to disturbances in mineral metabolism (Ao et al., 2017; Thumbigere-Math et al., 2018). Phospho1, a phosphatase, functions in skeletal and dental mineralization by initiating the deposition of hydroxyapatite within the lumen of matrix vesicles. In *Phospho1*-deficient mice, the formation and mineralization of acellular cementum remained normal, but cellular cementum showed enhanced deposition alongside delayed mineralization and the presence of cementoid (Zweifler et al., 2016).

Current evidence also highlights the regulatory role of E3 ubiquitin ligase in cellular cementum matrix apposition. A recent study revealed that Murine Double Minute 2 (Mdm2)-mediated p53 degradation enhances cementocyte viability, and these cementocytes subsequently regulate cementoblast activity and osteoclast function through a paracrine mode to maintain cellular cementum volume (Tian et al., 2025).

In the process of cementum matrix apposition, matrix proteins are involved in regulating cementum integrity. Matrix proteins such as Bone morphogenetic protein 2 (BMP2), cementum attachment protein (CAP), and cementum protein 1 (CEMP1) are crucial in initiating mineralization and cell recruitment, as well as endowing cells with cementogenesis potential (Hong et al., 2022). Bone sialoprotein (*Bsp*) deficiency impairs acellular cementum mineralization and results in subsequent root resorption (Foster et al., 2013). BMP1, a proteinase involved in extracellular matrix formation, is essential for cementum homeostasis *via* maintaining appropriate levels of procollagen I (Wang J. et al., 2017; 2021).

In addition, extracellular matrix protein such as Pentraxin 3 (*Ptx3*), has been reported as a positive regulator for cementum formation. Ptx3 deficiency restrained the formation of both acellular and cellular formation by inhibiting cementoblast differentiation (Wang S. et al., 2024).

## 3 Role of periodontal microenvironment in cementum destruction and regeneration

Cementum serves as a critical anti-resorptive barrier protecting tooth roots, primarily due to its inherent lack of remodeling activity (Iglesias-Linares and Hartsfield, 2017). The maintenance of cementum homeostasis is vital for ensuring long-term tooth stability and function.

**FIGURE 3:**
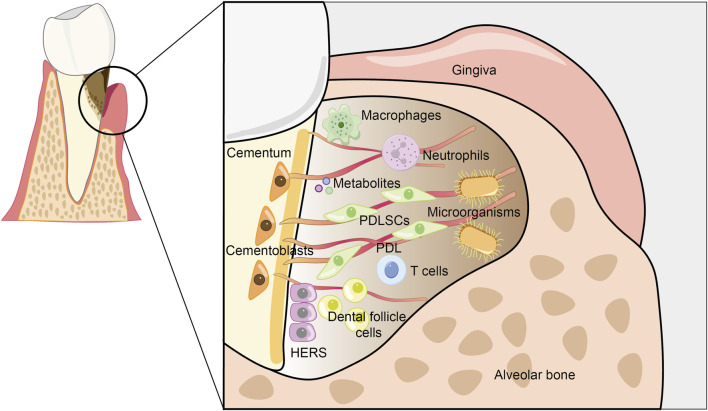
Schematic of the periodontal microenvironment. Host cells, microorganisms, metabolites, and other components collectively form a complex periodontal microenvironment. Bacteria are capable of secreting virulence factors, which activate the host’s immune response and ultimately lead to the destruction of periodontal tissues. Both bacteria and host cells, including cementoblasts, periodontal ligament stem cells, immune cells, *etc.*, release a diverse range of metabolites. These metabolites accumulate substantially in the local microenvironment, subsequently bringing about remarkable changes in the physical, chemical, and biological properties of the microenvironment. This, in turn, regulates the destruction of periodontal tissues and the regeneration of cementum.

Both physiological and pathological factors can lead to the destruction of cementum. Physiological root resorption is a normal phenomenon associated with tooth shedding, whereas pathological root resorption can compromise the structure and function of both cementum and dentin. Cementum resorption is typically induced by periodontal disease, chronic apical periodontitis, and excessive mechanical forces, such as those encountered during orthodontic tooth movement, as well as by tumors, trauma, cementum tears, cementum caries, and the replantation or eruption of teeth (Fuss et al., 2003; Sato et al., 2022). The destruction of cementum significantly impacts the integrity and functionality of periodontal tissues, thus cementum regeneration is regarded as the gold standard for periodontal regeneration (Arzate et al., 2015). Regenerative strategies employing mesenchymal stem cells (MSCs) have emerged as the predominant approach in cementum regeneration. However, the complex microenvironment presents challenges that require further investigation (Figure 3).

### 3.1 Host cells

#### 3.1.1 Stem cells

To date, research on the regulation of cementum regeneration by host cells has predominantly focused on stem cells and immune cells. The cementoblast mineralization model is extensively utilized as a framework for examining the mechanisms and outcomes associated with cementum regeneration. Cementoblasts secrete a mineralized matrix enriched with non-collagenous proteins, thus playing a crucial role in the restoration of cementum and the subsequent reconstruction of functional periodontal tissue (Nuñez et al., 2019). Periodontal ligament stem cells (PDLSCs) highly express mesenchymal stem cell surface markers and have multipotent differentiation capacity. With particular induction, it is capable of differentiating into cementoblast-like cells that produce mineralized nodules and express cementum-specific markers such as cementum protein 1 (CEMP1) and cementum attachment protein (CAP). Moreover, PDLSCs can create collagen fibers that form alveolar bone and cementum (Shinagawa-Ohama et al., 2017). Another group of mesenchymal progenitor cells surrounding the tooth germ, dental follicle cells (DFCs), are responsible for cementum, periodontal ligament, and alveolar bone formation in tooth development and periodontal regeneration (Zhou et al., 2019). It has the same embryonic origin as cementoblasts and robust osteogenic potential (E et al., 2021). Additionally, the co-culture of HERS cells with DFCs initiated epithelial-mesenchymal interaction (EMI) at the initial stage of *in vitro* culture, which facilitated the differentiation of osteoblasts/cementoblasts and played a role in repairing periodontal tissues (Bi et al., 2022).

#### 3.1.2 Immune cells

Immune cells dynamically shape the periodontal microenvironment by balancing pro-inflammatory and regenerative signals. Macrophages are pivotal regulators. M1 macrophages secrete TNF-α and IL-1β, which exacerbate osteoclast activation and bone resorption in periodontitis. Conversely, M2 macrophages promote tissue repair via anti-inflammatory cytokines (IL-10, TGF-β) and growth factors (VEGF, BMP-2) (Locati et al., 2020). Notably, macrophage depletion significantly reduces *Porphyromonas gingivalis*-induced alveolar bone loss (Lam et al., 2014). Our group has revealed that M2 macrophages have positive effects on cementoblasts by targeting inflammation and promoting mineralization (Huang et al., 2023).

T lymphocytes are an essential component of the adaptive immune system. T cell subsets also critically influence cementum regeneration. CD8^+^ T cells could enhance cementoblast differentiation (Lyu et al., 2019). During the process of periodontal regeneration, a reduction in T helper cells (Th17 cells) and an increase in regulatory T cells (Treg) are accompanied (Liu et al., 2024). This phenomenon might be attributed to the fact that Th17 cells secrete pro-inflammatory factors, which induce the apoptosis of PDLSCs and subsequently result in tissue destruction. On the other hand, Treg cells secrete anti-inflammatory factors such as IL-10 and TGF-β. These factors maintain the homeostasis of the periodontal microenvironment, thereby facilitating the differentiation of PDLSCs (Liu G. et al., 2025). Therefore, the balance between Th17 and Treg cells is crucial in determining regenerative outcomes. A predominance of Treg cells fosters an anti-inflammatory environment conducive to regeneration, while an excess of Th17 cells hinders cementum repair by maintaining an inflammatory state and inhibiting stem cell differentiation ability.

Although there is no direct evidence suggesting that neutrophils are involved in the regulation of cementum regeneration, they constitute an important part of the inflammatory periodontal niche. Neutrophils induce endoplasmic reticulum stress in Gli1^+^ stem cells through neutrophil extracellular traps (NETs) during periodontitis development, which in turn blocks their osteogenic differentiation and leads to the deterioration of alveolar bone (Cai et al., 2024). Their role is thus primarily indirect, modulating the broader stromal environment and affecting the regenerative capacity of progenitor cells.

In summary, stem cells and immune cells do not operate independently; rather, they engage in dynamic crosstalk that significantly influences the regenerative outcomes of cementum. Stem cells, including PDLSCs and DFCs, serve as the cellular foundation for cementogenic differentiation and the formation of new matrix. Concurrently, immune cells, particularly M2 macrophages and regulatory T cells (Tregs), modulate the inflammatory environment and foster a pro-regenerative milieu through the secretion of cytokines and growth factors. This orchestrated interaction between stem cells and immune cells is instrumental in restoring periodontal homeostasis and enhancing both the structural and functional regeneration of cementum.

### 3.2 Microbial communities

Dysbiotic oral microbial communities directly modulate the cementum regeneration. *Porphyromonas gingivalis* (*P. gingivalis*), as a key periodontal pathogen, has attracted extensive attention due to its inhibitory effect on cementum regeneration through multiple mechanisms. First, our group found that *P. gingivalis* inhibits cementoblast regeneration by directly impairing cementoblast mineralization and osteogenic/cementogenic differentiation of periodontal ligament cells (Ma et al., 2019; Huang X. et al., 2022). Moreover, *P. gingivalis* lipopolysaccharide (LPS) promotes macrophage M1 polarization, thus leading to bone tissue destruction (Dou et al., 2024). This mechanism indicates that *P. gingivalis* indirectly inhibits cementum regeneration by affecting immune cell functions. *Fusobacterium nucleatum* is yet another crucial pathogenic bacterium associated with periodontitis. Research indicates that oral gavage of *F. nucleatum* can lead to cementum destruction in mice (Lemaitre et al., 2017). The presence of an active herpesvirus infection during root formation hinders cementum development, and at puberty, the reactivation of the virus prompts bacterial pathogens to grow, causing rapid periodontal destruction in teeth with a compromised periodontium (Slots and Rams, 2024).

Additionally, a growing body of research has started to concentrate on the effects of bacterial outer membrane vesicles (OMVs) on periodontal regeneration. The interaction between pathogen-associated molecular patterns (PAMPs) on OMVs and pattern recognition receptors (PRRs) on host immune cells can lead to the activation of inflammatory responses and the initiation of adaptive immune responses (Zhang et al., 2025). OMVs from *Aggregatibacter actinomycetemcomitans* can transfer bioactive virulence factors to human gingival fibroblasts, consequently altering the progression of periodontal diseases. OMVs from *F. nucleatum* can cause macrophages to change from the M0 to the M1 phenotype, resulting in the loss of periodontal tissue in mice (Wang J. et al., 2024). Given the immunomodulatory properties of OMVs, researchers have suggested their potential application as vaccines. Studies have demonstrated that OMVs derived from *P. gingivalis* exhibit robust immunogenic and antigenic characteristics. In experimental models, mice immunized intranasally with a combination of OMVs and TLR3 agonist (Poly (I:C)), exhibited a marked decrease in the oral colonization of *P. gingivalis*, alongside increased levels of serum IgG in saliva (Nakao et al., 2011; 2016). Nevertheless, the specific mechanisms underlying the effects of OMVs on cementum destruction and regeneration warrant further investigation. As OMVs have the capacity to transport a variety of bioactive molecules, including proteins, lipids, and nucleic acids, future research could prioritize the comprehensive profiling of OMV cargos and the elucidation of their specific interactions with host signaling pathways, such as Toll-like receptors (TLRs) and inflammasomes, within the periodontal niche. This will be essential for designing precise molecular interventions aimed at inhibiting their pathological effects and leveraging their potential in regenerative medicine.

### 3.3 Metabolites

Microbe- and host-derived metabolites fine-tune immune responses and stem cell behavior. The subgingival microbiome produces a range of metabolic by-products during glucose and protein metabolism, including short-chain fatty acids (SCFAs), amines, various gases and other metabolites. This process accelerates the destruction of periodontal tissues and exerts a direct impact on host cells (Basic and Dahlén, 2023).

#### 3.3.1 Short-chain fatty acids (SCFAs)

SCFAs, such as butyrate and isovalerate, are primarily produced by microbial fermentation and show marked elevation in the saliva of patients with periodontal diseases (Yu et al., 2014). Systematic reviews indicate that SCFAs negatively impact epithelial cell viability by activating cellular events, including apoptosis, autophagy, and pyroptosis (Magrin et al., 2020). Among short-chain fatty acids, butyric acid is currently the fatty acid that has been studied most extensively. Evidence indicates that butyrate may have a detrimental impact on cells in periodontal tissue. Metabolomic analyses have revealed elevated concentrations of butyrate in the saliva of patients with periodontitis (Na et al., 2021). It has been reported that sodium butyrate inhibits osteogenesis in human PDLSCs by suppressing *Smad1* expression (Hou et al., 2022). Similarly, sodium butyrate activates the extrinsic and intrinsic apoptotic processes in murine cementoblasts (Lo et al., 2025). In contrast, butyrate inhibits the maturation of dendritic cells *in vivo*, suppresses local inflammatory infiltration, and ultimately reduces bone resorption in periodontitis models (Wu et al., 2023). Another key short-chain fatty acid, isovaleric acid, can be produced through the fermentation of a leucine-rich diet by subgingival microbes, including *P. gingivalis*. Within hPDLSCs, isovaleric acid elevates the levels and activities of gelatinases, including MMP2 and MMP9 (Zheng et al., 2025).

#### 3.3.2 Lactate

Lactate exemplifies a metabolite with a complex, context-dependent function. It is produced not only by bacteria but also by host cells during processes like glycolysis, which complicates its role (Ishikawa et al., 2021). The mineralization process of cementoblasts accompanies an increase in lactate production, suggesting a potential role in anabolism (Wang et al., 2023) However, lactate inhibits the osteogenic differentiation of human PDLSCs (Luo et al., 2022), highlighting a stark contrast between its effects in different cell types and microenvironments.

#### 3.3.3 Gases

Apart from short-chain fatty acids, hydrogen sulfide (H_2_S), methyl mercaptan (CH_3_SH), and various dimethyl sulfides collectively account for approximately 90% of the volatile sulfur compounds (VSCs) found in the oral cavity. These compounds are indicators of periodontal disease (Miura et al., 2025). H_2_S, as a typical metabolite of periodontal pathogens, facilitates bacterial survival and proliferation, thus exacerbating periodontitis development and periodontal tissue destruction (Xie et al., 2025).

#### 3.3.4 Vitamins and other metabolites

Currently, research regarding the regulation of cementum destruction and regeneration by metabolites remains scarce. Increased vitamin D drives the tooth and cementum resorption signaling through the vitamin D receptor in cats (Booij-Vrieling et al., 2010). IL-1alpha can induce Prostaglandin E2 production in murine cementoblasts through a time-dependent fashion, which is a bioactive metabolite of arachidonic acid (Noguchi et al., 2007).

Overall, current research endeavors regarding metabolites predominantly center around their influence on the progression of periodontitis inflammation. In contrast, research into the regulation of periodontal regeneration by metabolites, particularly their potential to be harnessed for therapeutic benefit, remains relatively scant and represents a promising frontier for future investigation. Future studies should prioritize comprehensive metabolite profiling in clinically relevant models to identify key regulatory molecules, and explore the therapeutic potential of targeted metabolite delivery or blockade to precisely modulate the periodontal microenvironment and enhance cementum regeneration.

## 4 Mechanisms of periodontal microenvironment components in regulating cementum regeneration

The complexity of the periodontal microenvironment gives rise to diverse and intricate regulatory mechanisms for cementum regeneration. In the study of odontoblast regeneration mechanisms, traditional research has primarily focused on classical signaling pathways. However, with the development of high-throughput sequencing technologies such as transcriptomics and metabolomics, researchers have begun to emphasize the regulatory roles of metabolic pathways, organelle functions, and epigenetic modifications in cementum regeneration (Table 3).

**TABLE 3:** Mechanisms of cementum regeneration.

Type	Specific mechanism	Results	References
Signal transduction networks	ERK1/2 signaling pathway	Promoting periodontal regeneration.	Zhao et al. (2025)
	Wnt/β-catenin pathway	Promoting cementogenesis of PDLSCs and DPSCs.	Wu et al., 2022; Cheng et al., 2025b
	FOXO1 pathway	Driving the differentiation of dental pulp stem cells into cementoblasts.	Cheng et al. (2025a)
	NF-κB pathway	Activated under inflammation.	Nemoto et al., 2006; Zhang et al., 2020b
	MAPK signaling pathway	Promoting cementoblast mineralization.	Ma et al. (2019)
	AKT signaling pathway	Contributing to the restoration of cementum.	Huang et al. (2022b)
Organelle homeostasis and metabolic regulation	Glycolysis	Cementoblasts mainly utilize glycolysis during mineralization process.	Wang et al. (2023)
	Mitochondrial function	Enhancing mitochondrial function under inflammation facilitates cementogenesis.	Ma et al., 2019; Li et al., 2022b; Wang et al., 2022
	Endoplasmic reticulum stress	Adversely affecting cementum formation	Xu et al. (2023)
	Pyroptosis	*P. gingivalis* infection leads to pyroptosis	Peng et al. (2023)
	Autophagy	The influence on cementoblast mineralization remains debatable.	Liu et al., 2022a; Yong et al., 2022b
Epigenetic modification	miRNAs	miR-3064-3p, miR-361-3p, miR-181b-5p and miR-155-3p regulate cementoblasts differentiation.	Wang et al., 2017b, 2018, 2019; Liao et al., 2019
	Long noncoding RNAs	LncRNA-p21 downregulates cementoblasts mineralization.	Huang et al. (2022a)
	Circular RNAs	CircLRP6 positively regulates cementoblast differentiation.	Li et al. (2021)
	DNA demethylation	Tet1 negatively modulates *P. gingivalis*-triggered pyroptosis; *P. gingivalis* blocks DNA methylation in cementoblasts.	Peng et al., 2023; Liu et al., 2025b
	RNA methylation	RNA N6-methyladenosine demethylase promotes cementoblast mineralization.	Li et al., 2022a; Sun et al., 2022b
	Histone demethylation	Promoting cementoblast mineralization.	Yang et al. (2025)
	Acetylation	Sirtuin 3 rescues *P. gingivalis*-impaired cementogenesis *via* SOD2 deacetylation.	Huang et al. (2025)
	Lactylation	HADHA lactylation promotes cementoblast mineralization.	Yang et al. (2025)

### 4.1 Signal transduction networks

The development and regeneration of cementum are precisely regulated by an intricate network of signaling pathways that coordinate cellular differentiation, matrix deposition, and mineralization. This signal transduction network exerts a powerful influence on cementogenesis. The major signaling systems involved can be functionally categorized into regeneration-associated pathways, inflammation-regulating pathways, and immune-modulating pathways, each contributing uniquely to cementum formation and maintenance.

#### 4.1.1 Regeneration-associated signaling pathways

Several key pathways directly promote cementogenic differentiation and matrix formation. Wnt/β-catenin signaling is a central regulator of cementum matrix apposition. Wnt3a could improve the efficacy of DPSCs-mediated cementum regeneration through activating the canonical Wnt pathway (Cheng et al., 2025b). Connective tissue growth factor facilitates the repair of cementum and cementogenesis of PDLSCs *via* the Cx43/β-catenin axis (Wu et al., 2022). The FOXO1 pathway is mediated by WNT3A to drive the differentiation of dental pulp stem cells into cementoblasts, thereby supporting the formation and preservation of cementum tissue (Cheng et al., 2025a). Additionally, metformin promotes periodontal regeneration through the ERK1/2 signaling pathway, a non-AMPK pathway (Zhao et al., 2025).

#### 4.1.2 Inflammation-regulating pathways

Under inflammatory conditions, various kinases and immune-related signaling pathways play crucial roles in regulating cementoblast function and cementum regeneration. NF-κB pathway activity is increased under inflammatory stimulation. YAP enhances the mineralization of cementoblasts that have been transiently treated with TNF-α, in part by decreasing the activity of the NF-κB pathway (Zhang L. et al., 2020). In addition, our group determined that the MAPK signaling network adjusts the supportive effects of CXXC5 in the mineralization process of cementoblasts (Ma et al., 2019). Ckip-1 inhibits inflammation and enhances cementoblast mineralization through MAPK and AKT pathways, potentially contributing to the repair of cementum affected by *P. gingivalis* (Huang X. et al., 2022).

#### 4.1.3 Immune-modulating pathways

Immune regulatory pathways fine-tune the response of cementogenic cells to pathogens and inflammatory stimuli. Nucleotide-binding oligomerization domains (NODs) and toll-like receptors (TLRs) are functionally expressed in human cementoblasts and can trigger innate immune responses (Ahn et al., 2013). In particular, TLR-2 mediate bacterial stimulation; *P. gingivalis* lipopolysaccharide significantly induced transcripts of NF-κB. and the activation of NF-κB was inhibited after pre-treated with anti-TLR-2 (Nemoto et al., 2006). Additionally, Hypoxia-inducible factor (HIF)-1α plays roles in the immune response of cementoblasts *via* immune-inhibitory PD-L1 (Yong et al., 2022a). However, the research on the influence of immune responses on the functions of cementoblasts still remains at the phenotypic level, and the underlying mechanisms need to be further explored in depth.

### 4.2 Organelle homeostasis and metabolic regulation

As previously described, the periodontal microenvironment harbors abundant metabolites. Organelles such as mitochondria concentrate metabolic precursors and participate in both anabolic and catabolic processes. This mechanism involves complex interactions between organelles and the regulation of metabolic pathways. The mitochondrial associated membrane arrays (MAMs), contact sites between mitochondria and the endoplasmic reticulum, play a crucial role in the exchange of metabolites and calcium ions, which is vital for maintaining cellular and organ homeostasis (Theurey and Rieusset, 2017; Jain and Zoncu, 2022). Additionally, interactions between mitochondria and other organelles like peroxisomes and lysosomes also significantly contribute to metabolic regulation. These interactions modulate cellular metabolic states through metabolic signaling and reverse signaling pathways (Kim and Bai, 2022).

During cementum regeneration, alterations in the metabolic microenvironment and organelle homeostasis significantly influence cellular development and function. Our group noticed a marked rise in glucose consumption and lactate production, along with an elevated expression of genes related to glycolysis. This indicates that glycolysis positively regulates cementoblast mineralization (Wang et al., 2023). *Porphyromonas gingivalis* infection can affect cellular glycolysis and subsequently lead to pyroptosis. Inhibition of glycolysis enhances the viability of cementoblasts and diminishes pyroptosis. This result is contrary to that under mineralization conditions and may be related to the time and concentration of bacterial stimulation (Peng et al., 2023). In addition to glycolysis, mitochondrial ROS also participates in *P. gingivalis-induced* pyroptosis in cementoblasts (Sun et al., 2024). Upon exposure to external stimuli, the functions of organelles and metabolic states undergo alterations. CXXC5 alleviates the suppression of cementogenesis by *P. gingivalis* by promoting mitochondrial biogenesis (Ma et al., 2019). Inflammatory tissues exhibit hypoxia, which induces impairments in mitochondrial biogenesis and thereby suppresses the mineralization of cementoblasts (Wang et al., 2022). Enhancing the mitochondrial function of cells under an inflammatory stimulus can facilitate the cementogenesis of PDLSCs (Li X. et al., 2022). Beyond mitochondria, another organelle, the endoplasmic reticulum (ER), also participates in the regulation of cementum regeneration. Spliced X-box binding protein 1, which is produced by ER stress, adversely affected cementum formation (Xu et al., 2023).

The role of autophagy in cementum regeneration remains a subject of ongoing debate. Triggering autophagy induction is accompanied by a decline in cementoblast mineralization function (Yong et al., 2022b). However, in a mouse orthodontic tooth movement model, rescuing the impeded autophagy process in cementoblasts can enhance cementogenesis and alleviate orthodontic force-induced root resorption (Liu H. et al., 2022).

### 4.3 Epigenetic modifications

Epigenetic processes, including non-coding RNA expression, DNA methylation, RNA modification, histone modifications, and act coordinately to regulate cellular differentiation and homeostasis (Nazor et al., 2012). This complex and multi-level process can affect cementum regeneration by regulating gene expression and cell fate.

#### 4.3.1 Non-coding RNAs

Over the past few years, studies have predominantly centered on the regulation of cementum regeneration by non-coding RNAs, including miRNAs, lncRNAs, and circRNAs. Our group performed microarray and screening analyses of miRNAs during cementoblast mineralization. The results indicate that miR-3064-3p and miR-361-3p regulate cementoblast differentiation by targeting *Dkk1* and *Nfat5*, respectively (Wang et al., 2018; Liao et al., 2019). When cementoblasts are in an inflammatory state induced by TNF-α, miR-155-3p inhibited cementoblast mineralization by suppressing *Kctd1*, and miR-181b-5p moderates TNF-α-induced cementoblasts inflammatory response by inhibiting *IL-6* (Wang X. et al., 2017; 2019). Furthermore, evidence suggests that long noncoding RNAs and circular RNAs (circRNAs) play critical regulatory roles in cementoblasts differentiation. LncRNA-p21 downregulated the expression of mineralization-related markers of cementoblasts (Huang H. et al., 2022). CircLRP6 positively regulates cementoblast mineralization (Li et al., 2021).

#### 4.3.2 DNA and RNA modifications

DNA and RNA modifications play crucial roles in gene expression regulation. DNA modifications mainly include DNA methylation and demethylation, which can modify genes to silence or activate them by influencing methylation states in the promoter regions (Horii et al., 2020). RNA modifications, particularly N6-methyladenosine (m6A) modification, can affect RNA stability, translation efficiency, and degradation (Frye et al., 2018). Our group has discovered that DNA demethylase, Tet methylcytosine dioxygenase 1, negatively modulates *P. gingivalis*-triggered pyroptosis (Peng et al., 2023). Moreover, global DNA methylation was upregulated during cementoblast mineralization. *Porphyromonas gingivalis* inhibits DNA methylation by suppressing Dnmt3a in cementoblasts, leading to mitochondrial-dependent apoptosis and resulting in disrupted cementogenesis. Contract with this finding, prolonged LPS stimulation causes DNA methylation of GJA1, BMP2, and BMP4 in human cementoblasts (Liu H. et al., 2025). These apparently conflicting observations regarding DNA methylation under bacterial challenge may arise from differences in experimental conditions, including cell type specificity, duration of stimulation, and pathogen load. Thus, the function of DNA methylation in cementoblast regeneration remains further investigation. Fat mass and obesity-associated protein (Fto), a kind of RNA N6-methyladenosine demethylase, has been found promoting cementoblast mineralization under normal and inflammatory conditions (Li B. et al., 2022; Sun Q. et al., 2022).

#### 4.3.3 Post-translational modifications (PTMs)

Post-translational modifications have emerged as a prominent field in research regarding cellular differentiation, development, and regeneration. PTMs occur in both histones and non-histone proteins (Shvedunova and Akhtar, 2022). It includes acetylation, lactylation, and other modification types, which can alter chromatin structure and regulate gene expression (Li et al., 2025). Sirtuin 3, a NAD^+^-dependent protein deacetylase, can rescue *P. gingivalis*-impaired cementogenesis *via* SOD2 deacetylation (Huang et al., 2025). Lysine (K)-specific demethylase 6B regulates HADHA by mediating histone demethylation and lactylation, thereby upregulating FAO and thus promoting mineralization (Yang et al., 2025). In summary, epigenetic modifications present a novel perspective for investigating cementum regeneration. Nevertheless, to date, relatively few studies have explored whether epigenetic modifications can modulate the cementogenic differentiation of PDLSCs. Thus, further in-depth research in this area is warranted.

## 5 Cementum regeneration strategies based on the cementum development and periodontal microenvironment

**FIGURE 4:**
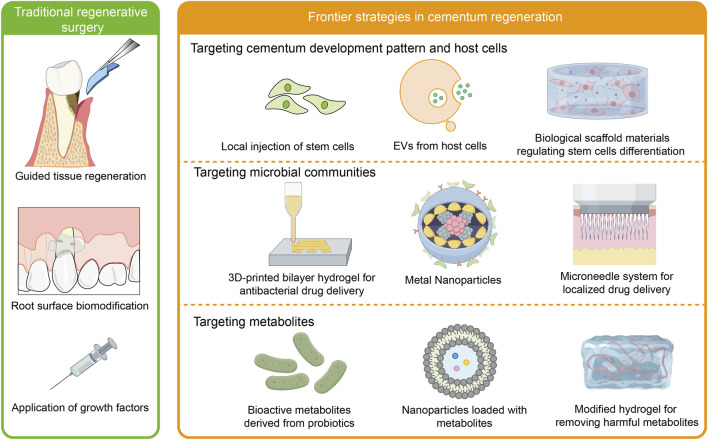
Cementum regeneration strategies. Traditional methods for periodontal regeneration, such as GTR, root surface biomodification, and application of growth factors, face multiple clinical challenges, particularly in achieving complete cementum regeneration. Biomaterials targeting host cells, microorganisms, and metabolites in the periodontal microenvironment have emerged as a promising approach for achieving functional regeneration of cementum.

Cementum regeneration is crucial for restoring periodontal function. While traditional approaches like guided tissue regeneration (GTR) have shown some clinical efficacy, they still fall short in fully restoring cementum functions. This has driven growing research into microenvironment-based regenerative methods, aiming to achieve more effective periodontal tissue regeneration (Figure 4).

### 5.1 Histological assessment of cementum regeneration

The primary outcome measures of cementum regeneration were the cementum measurements (i.e., length, thickness, or regeneration percentage of newly-formed cementum). Hematoxylin and Eosin (H&E) staining is highly applied in the vast majority of cases; however, other staining techniques were also used—Toluidine blue staining, Azan staining and Levai-Laczkó staining. Toluidine blue staining is recognized for its ability to more clearly indicate the height of cementum. Azan staining in demonstrating newly formed collagen and cementum. The Levai-Laczko staining technique is employed to measure the length of newly formed cementum (Crossman et al., 2018). Concrete techniques are as follows: 1) measuring the length of newly formed cementum (%) (sum of individual lengths divided by total length before the creation of defect); 2) measuring newly-formed cementum thickness (mm); 3) measuring the proportion of the newly-formed cementum (%) (distance between apical side and coronal side of newly-formed cementum was divided by defect height).

A favorable cementum regeneration exhibits a functionally oriented PDL of relatively high density inserted into newly formed cementum. Histology and histomorphometry can be applied to identify cementoblasts and cementocytes, evaluate the orientation and density of the PDL fibers, as well as analyze PDL fiber orientation from the cementum to bone and other tissue characteristics (Wikesjö et al., 2004; Iwata et al., 2009; Padial-Molina et al., 2015).

### 5.2 Traditional regenerative surgery

Periodontal treatment can promote periodontal healing (Goldstein et al., 1996). One of the goals of current regenerative surgical approaches is to prevent the growth of connective tissue cells, such as epithelium and gingiva, towards the root surface by placing a barrier membrane between the soft tissue of the gingiva and the root of the affected tooth, thus allowing periodontal ligament cells with periodontal regenerative capacity to occupy the root surface.

Based on this biological concept, researchers have devised several additional treatment options to improve the success and predictability of regenerative procedures. These include guided tissue regeneration, root surface biomodification, and the use of a range of growth factors. Guided tissue regeneration, where bioresorbable membranes and bone grafts are used to guide and control periodontal tissue regeneration (Al-Hamdan et al., 2003; Reynolds et al., 2003; Sheikh et al., 2017). In a study, enamel matrix derivatives, GTR, and their combination treatments were more effective than flap surgery, while the differences across regeneration therapies remained minor (Sj et al., 2020). Utilization of β-TCP and platelet-rich plasma may facilitate membrane integration and periodontal regeneration (Dőri et al., 2022). Although GTR offers great therapeutic effects, it has limitations as a periodontal regeneration therapy, including membrane collapse in larger defects, incomplete tissue integration, limited osteogenic and cementogenic ability of commercially available membranes, lack of anti-inflammatory and antimicrobial properties (Ul Hassan et al., 2021). Thus, membranes based on stem cells, scaffolds, and bioactive compounds have emerged rapidly, and have promising applications (Liang et al., 2020).

Root surface biomodification, which increases the migration and attachment of mesenchymal cells to the exposed root surface. When root surface treatment is performed using acids, particularly citric acid and tetracycline. These root conditioners accelerate osteogenesis, enhance bone deposition and connective tissue attachment, and promote better clinical outcomes (Mariotti, 2003; Damante et al., 2019). Some authors have utilized root lasers, such as Er, Cr: YSGG laser and Er, YAG laser, to optimize the healing process and raise the success rate of root coverage (Eltayeb et al., 2021; Tunar et al., 2021).

The use of a range of growth factors, including platelet-derived growth factor, insulin-like growth factor-1, bone forming protein 2, transforming growth factor beta, basic fibroblast growth factor and enamel matrix protein, can promote periodontal membrane and bone regeneration (Yu et al., 2016; Mikami et al., 2022). Currently, platelet concentrates, including platelet-rich plasma, platelet-rich fibrin, and concentrated growth factor, are being utilized extensively for sinus floor elevation, alveolar ridge preservation, periodontal bone defect, and gingival recession. Particularly, it is believed that platelet extracellular vesicles are crucial for PC-induced tissue regeneration (Sun J. et al., 2022). Despite the effectiveness of these regimens, the predictability of treatment is limited, and the ability to fully restore the original periodontal membrane structure, particularly in terms of bone regeneration, is very limited.

### 5.3 Frontier strategies in cementum regeneration

#### 5.3.1 Targeting cementum development pattern and host cells

Regenerative strategies for cementum increasingly leverage insights from developmental biology and host cell manipulation to achieve functional restoration. These approaches aim to recapitulate key processes in cementogenesis by targeting the differentiation of host cells, modulating the local microenvironment, and providing appropriate biological cues for tissue formation. In recent years, matrix proteins, stem cells, extracellular vesicles, and biomaterial scaffolds have demonstrated significant potential in cementum regeneration *via* modulating the function of host cells.

#### 5.3.2 Matrix protein-based regenerative strategies

As previously described, matrix proteins are implicated in cementum development and the maintenance of cementum homeostasis. Ding et al. developed a core/shell fibrous super-assembled framework (SAF)-based sequential growth factor delivery system that is designed to release basic fibroblast growth factor (bFGF) and BMP-2 sequentially. bFGF, as a potent mitogen, primarily promotes the proliferation and migration of human PDLSCs, thereby expanding the progenitor cell pool available for regeneration. Subsequently, BMP-2 induces osteogenic/cementogenic differentiation of these cells by activating SMAD-dependent signaling pathways and upregulating key mineralization-related genes, including Runx2, Osterix, and osteocalcin. This delivery system significantly enhanced the new bone formation and regeneration of periodontal and cementum *in vivo* (Ding et al., 2020).

#### 5.3.3 Stem cells

Local injection of BMSC might limit the inflammation of periodontitis tissue and facilitate the regeneration of periodontal tissue (Lu et al., 2020). Combining a xenogeneic collagen matrix (CMX) with gingiva-derived mesenchymal cells (GMSCs) resulted in the formation of new cementum (Sanchez et al., 2022). Research indicates that dental pulp mesenchymal stem cells (DPSCs) can be induced to express key osteogenic transcription factors and promote the secretion of type I collagen and osteopontin. This process accelerates the deposition of mineralized matrix (Ching et al., 2017). Besides, DPSCs cultured on cementum surfaces can create a matrix resembling cementoid (Mata et al., 2022). In accordance with this, A method involving the injection of human dental pulp stem cells (hDP-MSC) was designed to support periodontal regeneration in a non-invasive way, which can ameliorate attachment loss (Liu Y. et al., 2025). Furthermore, researchers have discovered that PDLSCs and DPSCs can self-assemble into structures resembling natural tooth roots in the absence of scaffolds. This self-assembly capability enables PDLSCs and DPSCs to play a crucial role in forming dentin-pulp and PDL-cementum organoids (Li et al., 2020; Calabrese et al., 2024).

Despite promising preclinical results, the clinical translation of stem cell-based therapies for cementum regeneration faces several significant challenges. Substantial variability in therapeutic outcomes across studies, often attributed to differences in cell isolation methods, culture conditions, and donor heterogeneity, has hampered clinical standardization. Addressing these translational challenges through standardized protocols, rigorous clinical trials, and mechanistic studies will be essential for realizing the full clinical potential of stem cell-based cementum regeneration.

#### 5.3.4 Extracellular vesicles

Extracellular vesicles, serving as crucial mediators of intercellular communication, contain cargoes such as lipids, mRNA/miRNA, and proteins (Bonsergent et al., 2021). Small extracellular vesicles from LPS-pretreated dental follicle cells promote periodontal regeneration in inflammation (Shi et al., 2020). In *P. gingivalis*-driven inflammatory environments, M2-exosomes (M2-EXO), derived from genetically engineered M2 macrophages, have been demonstrated to activate cementoblast mitochondrial biogenesis through Let-7f-5p miRNA-mediated targeting and silencing of Ckip-1, ultimately driving cementum formation (Huang et al., 2024). Extracellular vesicles from compression-loaded (Comp-EVs) could promote macrophage M2 polarization and significantly enhance the clearance of apoptotic cells. The local administration of Comp-EVs alleviates cementum damage in a mouse root resorption model by promoting macrophage-mediated tissue repair (Yang et al., 2024). In a periodontal bone defect model, local injection of apoptotic extracellular vesicles derived from human dental pulp stem cells aids in the renewal of periodontal tissue regeneration, especially reconstruction of cementum (Xia et al., 2025).

Although EV-based therapies show promise, significant challenges hinder their clinical adoption. Scalability is a key issue, as current production methods cannot consistently produce the necessary quantities for clinical use. Additionally, the variability in EV cargo, even from similar cell types and conditions, complicates standardization and quality control. Furthermore, issues with targeted *in vivo* delivery—such as rapid clearance, poor tissue targeting, and limited penetration into disease sites—diminish therapeutic effectiveness, requiring improved delivery strategies.

#### 5.3.5 Biological scaffold materials

Biological scaffold materials provide structural support and bioactive stimulation in periodontal tissue engineering, particularly nanofibrous membranes and modified hydrogels (Zhou et al., 2021). Liu et al. achieved the rapid release of IL-2 and TGF-β and long-term release of miR-10a by preparing nanofibrous sponge microspheres to regulate T cells and reduce the secretion of inflammatory cytokines, thus rescuing the loss of periodontal bone tissue in the mouse periodontitis model. This immunomodulatory design demonstrates particular relevance for inflammatory conditions, as it actively promotes a regenerative immune microenvironment despite pathological challenges (Liu et al., 2018). Strontium-containing mesoporous bioactive glass (Sr-MBG) scaffolds have been demonstrated to significantly stimulate osteogenic/tooth-related gene expression in periodontal ligament cells (PDLCs), while maintaining excellent apical prismatic mineralization capacity under simulated body fluid environments (Chen et al., 2016). A titanium nanosurface that mimics a biological microenvironment can promote the natural regeneration of cementum and other periodontal tissues (Yamada et al., 2022). Through hydrogel design, sustained drug release can be achieved to enhance therapeutic efficacy. For instance, the photopolymerized GelMA composite hydrogel containing ZIF-8 continuously releases zinc ions, promoting osteogenic differentiation of bone marrow mesenchymal stem cells. Besides, it can reduce the bacterial load, relieve inflammation (Liu Y. et al., 2022). Three-layer nano-composite hydrogel scaffolds can simultaneously regenerate both hard tissues (cementum and alveolar bone) and soft tissues (periodontal ligament), achieving complete regeneration of periodontal defects (Sowmya et al., 2017).

#### 5.3.6 Targeting microbial communities

The application of antimicrobial strategies in promoting periodontal regeneration has become a crucial focus in contemporary periodontal disease treatment research. As a chronic inflammatory condition caused by bacterial infection, periodontal disease often fails to achieve long-term efficacy through conventional treatments like mechanical scaling and surgical interventions, with high recurrence rates. Mesoporous hydroxyapatite/chitosan loaded with recombinant human amelogenin inhibit the growth of periodontal pathogens and promote the formation of bone and cementum-like tissue (Liao et al., 2020). This indicates that antimicrobial biomaterials is a key research frontier in dental medicine, which can simultaneously enhance regenerative functions.

The study on hierarchical chitin nanocrystal-based 3D-printed bilayer hydrogel demonstrates an innovative dual-drug delivery platform that not only exhibits antibacterial properties but also promotes periodontal tissue regeneration. This bilayer membrane, combining lipid nanoparticle-loaded grape seed extract, simvastatin, and chitin nanocrystals, demonstrates excellent mechanical performance and sustained drug release capabilities. *In vivo* studies revealed significant effects in improving the recovery of alveolar bone and lowering inflammation (Dos Santos et al., 2024).

Furthermore, the introduction of zinc oxide nanoparticles has been proven to exhibit significant antibacterial and osteogenic-promoting effects in periodontal regeneration. Studies demonstrate that these nanoparticles effectively inhibit the growth of periodontal pathogens while promoting the expression of bone formation-related genes and proteins in both *in vitro* and *in vivo* experiments (Watanabe et al., 2012; Münchow et al., 2015). This multifunctional biomaterial provides new therapeutic approaches for periodontal disease management.

Additionally, the hyaluronic acid-based minocycline dissolution microneedle system has demonstrated potential in localized drug delivery. Through an innovative photoresistance strategy, this system significantly enhances the stability and antibacterial efficacy of minocycline, showing superior therapeutic effects compared to traditional treatments in *vivo* studies (Song et al., 2025). This novel localized drug delivery system provides a painless and efficient alternative for periodontal disease treatment.

In conclusion, antimicrobial strategies in periodontal regeneration not only directly inhibit bacterial growth but also indirectly improve periodontal health by promoting bone formation and tissue regeneration. These research findings provide a crucial theoretical foundation and technical support for developing more effective periodontal disease treatments in the future. However, most existing research focuses on alveolar bone regeneration and anti-inflammatory effects, with limited investigation into the direct impact on cementum regeneration. Future studies should specifically evaluate how these antimicrobial biomaterials influence cementoblast mineralization capacity and deposition of the cementum matrix. Clinical application faces challenges like ensuring long-term biocompatibility, especially with nano-materials, due to potential particle spread and tissue reactions. Precision in targeting antimicrobial effects without harming beneficial microbes is crucial. Additionally, scaling up production while ensuring consistency and sterility poses significant commercial challenges.

#### 5.3.7 Targeting metabolites

Recent years have witnessed significant advancements in the field of metabolite-mediated periodontal regeneration, particularly in utilizing metabolic compounds to regulate inflammation and promote tissue regeneration (Sui et al., 2024). The role of metabolites in periodontal regeneration primarily manifests through their regulation of cellular functions and enhancement of the microenvironment. For instance, bioactive metabolites derived from lactic acid bacteria have been found to support periodontal health by inhibiting oxidative processes and exerting antimicrobial effects, thereby maintaining periodontal homeostasis (Sulijaya et al., 2020). Furthermore, studies demonstrating that methotrexate-loaded yeast β-glucan-based nanoparticles can promote periodontal bone regeneration in inflammatory microenvironments reveal that these metabolites can transform macrophages from an inflammatory M1 phenotype to an anti-inflammatory M2 phenotype, effectively suppressing inflammation and repairing alveolar bone tissue (Chen et al., 2024).

Specifically eliminating harmful metabolites within the periodontal microenvironment while replenishing the metabolites essential for cell proliferation and differentiation can effectively facilitate periodontal regeneration. A kind of hyaluronic acid methacryloyl/ZnO (HMZ) composite hydrogel with an H_2_S-scavenging ability was able to remove H_2_S by a reaction with ZnO, thus achieving efficient periodontal bone regeneration (Xie et al., 2025). Alpha-ketoglutarate (AKG) is a key component of the tricarboxylic acid (TCA) cycle. Locally delivered cell-permeable AKG significantly promotes osteogenic differentiation and mouse bone regeneration, which shows great promise to improve cementum restoration and periodontal regeneration (Wang Z. et al., 2024). It is important to acknowledge that while several metabolite-targeting strategies show promise for periodontal regeneration, direct evidence of their specific effects on cementoblast mineralization and cementum matrix formation is still emerging.

In summary, the application of metabolites in periodontal regeneration has demonstrated their multifaceted roles in regulating inflammation, promoting cellular differentiation, and improving the microenvironment. While current evidence primarily supports their general periodontal regenerative effects, future research exploring metabolite-based strategies specifically targeting cementum regeneration represents a promising frontier in periodontal tissue engineering. Addressing the current limitations in delivery stability, metabolite half-life, and host response variability will be essential for translating these approaches to clinical practice.

## 6 Conclusion

Cementum regeneration represents a critical challenge and opportunity in periodontal tissue engineering. This review has synthesized key insights into the complex interplay between host cell dynamics, microbial communities, and metabolic factors that collectively influence the periodontal microenvironment and cementum homeostasis. We have highlighted how immune-stem cell crosstalk, epigenetic regulation, and microbial-metabolite interactions create permissive or inhibitory niches for regeneration. Particularly significant are the advancements in scaffold technologies that now incorporate immunomodulatory and antimicrobial features while promoting cementogenic differentiation.

A central insight emerging from this synthesis is the fundamental importance of acellular cementum in periodontal health. Unlike cellular cementum, acellular cementum provides the critical structural interface for periodontal ligament fiber anchorage, ensuring long-term tooth stability through its unique extracellular matrix composition and resistance to resorptive processes. Its non-remodeling nature and specific collagen organization make it essential for maintaining periodontal attachment throughout life.

Despite these advancements, substantial challenges persist. Present regenerative strategies primarily focus on the formation of cellular cementum, whereas the predictable regeneration of acellular cementum—characterized by its precise fibrous architecture and insertion sites—remains challenging. Future research should prioritize three critical directions. Firstly, it is imperative to elucidate the developmental mechanisms that govern the assembly of the acellular cementum matrix. Secondly, there is a need to develop biomaterials that accurately replicate its unique structural and functional properties. Thirdly, advancing clinical translation necessitates the establishment of standardized outcome measures specifically designed to assess functional periodontal attachment. The integration of single-cell technologies, advanced biomaterial design, and patient-specific approaches will be crucial for achieving comprehensive and functional periodontal regeneration.
